# Evasion of adaptive immunity by HIV through the action of host APOBEC3G/F enzymes

**DOI:** 10.1186/s12981-017-0173-8

**Published:** 2017-09-12

**Authors:** Michael Grant, Mani Larijani

**Affiliations:** Program in Immunology and Infectious Diseases, Division of Biomedical Sciences, Faculty of Medicine, Memorial University of Newfoundland, St. John’s, NF A1B 3V6 Canada

## Abstract

APOBEC3G (A3G) and APOBEC3F (A3F) are DNA-mutating enzymes expressed in T cells, dendritic cells and macrophages. A3G/F have been considered innate immune host factors, based on reports that they lethally mutate the HIV genome in vitro. In vivo, A3G/F effectiveness is limited by viral proteins, entrapment in inactive complexes and filtration of mutations during viral life cycle. We hypothesized that the impact of sub-lethal A3G/F action could extend beyond the realm of innate immunity confined to the cytoplasm of infected cells. We measured recognition of wild type and A3G/F-mutated epitopes by cytotoxic T lymphocytes (CTL) from HIV-infected individuals and found that A3G/F-induced mutations overwhelmingly diminished CTL recognition of HIV peptides, in a human histocompatibility-linked leukocyte antigen (HLA)-dependent manner. Furthermore, we found corresponding enrichment of A3G/F-favored motifs in CTL epitope-encoding sequences within the HIV genome. These findings illustrate that A3G/F‐mediated mutations mediate immune evasion by HIV in vivo. Therefore, we suggest that vaccine strategies target T cell or antibody epitopes that are not poised for mutation into escape variants by A3G/F action.

## Background

### The classical view of A3G/F as anti-viral host restriction factors and innate immune agents

APOBEC3G (A3G) and APOBEC3F (A3F) are members of the apolipoprotein B mRNA-editing enzyme catalytic polypeptide-like editing complex (APOBEC) family of cytidine deaminase enzymes. This family includes activation-induced cytidine deaminase (AID), APOBEC1, APOBEC2, APOBEC3A-H, and APOBEC4. All APOBEC3 (A3) sub-branch members (A3A, A3B, A3F, A3G and A3H) exhibit varying degrees of anti-viral restriction activity, though A3G and A3F are the most potent restrictors of HIV. For this reason, the mechanisms of A3G/F have been the subject of intense study over the last decade. From these studies, it is appreciated that A3G/F mutate cytidine (C) to uridine (U) in trinucleotide mutational hotspots (CCC—which is GGG on the coding strand sequence—and less often TCC for A3G; TTC for A3F) in the reverse transcribed DNA copy of HIV, registering as guanine (G) to adenine (A) substitutions in the plus strand DNA [1–6]. These mutations can trigger DNA degradation or lead to stop codons, frame shift or mutated viral proteins. In addition to hindering HIV through their mutational activities, A3G/F can also physically sequester the viral RNA, block reverse transcriptase (RT) and obstruct integration into the host cell genome [7–9].

Since their discovery early in the last decade, the prevalent view of A3G/F has been as anti-retroviral restriction factors. This is because, first A3G/F significantly diminishes viral propagation in several in vitro or ex vivo reporter systems of HIV infection [1, 3, 6, 10]. Second, in said reporter systems of HIV infection and in biochemical assays, A3G/F introduce high levels of G to A mutations in viral genomes, in the range of hundred mutations/genome/replication round, orders of magnitude higher than HIV’s own reverse transcriptase (RT), which introduces approximately one mutation in the 10 kb-long HIV genome per replication cycle [11]. Third, the expansion of a single APOBEC3 gene in mice to 7 in primates and the high divergence within A3 branch amongst primates have been suggested to be concomitant with the emergence of modern lentiviruses, and interpreted as evidence for the important role that this family of enzymes plays in anti-retroviral immunity [12].

A3G/F have been considered as innate immune agents, because they are constitutively expressed in CD4+ T lymphocytes, macrophages, and dendritic cells regardless of infection status, though inflammatory cytokines can upregulate their expression post-infection [13]. Second, they non-discriminately mutate any cytoplasmic DNA, without sequence or antigenic specificity. It is well established that in the long-term, it is adaptive immunity that controls HIV infection. The antibody and CTL responses are key: presence of broadly neutralizing antibodies and the robustness of anti-HIV CTL are the two cornerstones of a successful anti-HIV immune response, and correlate inversely with disease progression [14–21]. Thus, in the mainstream view of A3G/F as innate immunity’s anti-viral host restriction factors, A3G/F have been proposed to serve as a first line of defense to limit viral replication, whilst the HIV-specific adaptive immune responses of CTL and antibodies take weeks to months to ramp up to effective levels.

### A novel role for A3G/F beyond innate immunity and in adaptive immunity

In contrast to lethal mutation of HIV’s genome and its effective restriction by A3G/F in various in vitro cell culture-based assays of HIV infectivity, A3G/F activity in vivo only leads to low level non-lethal mutations of the viral genome. There are several reasons for this: first, the Virion infectivity factor (Vif) protein of HIV binds and targets A3G/F for degradation via a ubiquitin-dependent proteosomal pathway. Thus, when Vif is present, A3G/F effectiveness in viral restriction is severely diminished, and experimental systems where A3G/F can functionally restrict HIV use Vif-deficient viruses F [22–27]. Second, due to their highly charged surfaces and propensity for non-specific DNA/RNA or protein–protein binding, most cellular A3G/F are rendered inactive due to entrapment in mega-dalton sized ribonuclear cytoplasmic high-molecular-mass complexes (HMM) [28]. Third, even in cells where A3G/F introduce a high mutational load on the invading viral genomes, their final mutational signature in synthesized virions is much reduced due to the inherent selection that successive stages of viral replication impose for fit viral genomes [29].

Since most HIV genomes would only suffer low level A3G/F mutations levels, we and others proposed that non-lethal levels of A3G/F-induced mutations could impact adaptive immunity in HIV-infected individuals through modification of specific epitopes [30]. Two earlier studies had examined the role of APOBEC-induced mutations on recognition of HIV CTL epitopes, using a bioinformatics approach and a mouse model, respectively [31, 32]. The former study reported that approximately one-third of rapidly diversifying regions of HIV mediating CTL escape are embedded in A3G hotspots and suggested that A3G/F action may aid HIV by mutating its CTL epitopes towards immune evasion. In contrast, the latter study reported that A3G mutations enhanced the virus-specific CTL response through the introduction of premature stop codons in the HIV genome that cause the generation of truncated or misfolded proteins. In this study, Vif+ or Vif− HIV was produced in the presence or absence of A3G in a cell line and subsequently used to infect peripheral blood mononuclear cells (PBMCs) followed by assessing their susceptibility to being killed by CTL bearing transgenic peptide-specific T cell receptors. This was the first study to show that in principle, the impact of A3G/F mutations could extend to the adaptive immune realm. Nevertheless, whether this might be the case in HIV-infected humans had not been examined.

We sought to determine if A3G/F mutations play a role in adaptive immunity in HIV-infection. To this end, we measured CTL recognition of wild type and A3G/F-mutated epitopes in a cohort of HIV-infected individuals. First, we cataloged all CTL epitopes in HIV proteins using the experimentally-verified HIV molecular immunology database, and identified CTL epitopes whose encoding sequences contain A3G/F-mutable hotspots. Next, we synthesized wild type and A3G/F-mutated peptide CTL epitopes restricted to various Class I HLA molecules and evaluated CTL recognition of wild type and A3G/F-mutated CTL epitopes by CTL from HLA-matched subjects. We found that in contrast to control peptides mutated in sequences outside A3G/F hotspots, A3G/F-induced mutations overwhelmingly diminished CTL recognition of HIV peptides [33].

Two further key observations can be taken as evidence for the physiological significance of A3G/F-mediated CTL evasion by HIV. First, we found that HIV’s genome has enriched for A3G/F mutational hotspots in sequences encoding CTL epitopes, either located within more immunogenic polypeptides (Gag, Pol and Nef), or restricted to class I HLA molecules that present highly immunogenic epitopes (e.g. HLA-B57) [33]. Second, we observed strong HLA specificity: for epitopes restricted to HLA-B57 and A2, A3G/F mutations significantly decreased CTL recognition [34]. In contrast, A3G/F mutations enhanced the CTL recognition of most HLA-B35-restricted epitopes, and a very limited subset of epitopes restricted to HLA-A2, A3, B44 and B57. This HLA specificity, namely the prevalence of escape mutations linked to HLA alleles that present immunogenic CTL epitopes, such as HLA-B57, fits closely with the pattern of well-characterized CTL escape variants in the population [35, 36]. Thus, the evolution of the viral genome to position more A3G/F target hotspots in immunogenic CTL epitopes, and the HLA specificity strongly suggest that A3G/F-mediated CTL escape is a significant aspect of HIV infection in vivo.

### Conclusions and implications for vaccine design

It is likely that A3G/F do indeed impact adaptive immunity in HIV-infected individuals, and that A3G/F-mediated CTL escape is a significant phenomenon in HIV infection. We note that a parallel role for A3G/F in mediating drug resistance mutations of the viral genome has already been demonstrated. Together, these results indicate that A3G/F carry significant pro-retroviral activities.

It will be important to understand the extent of inter-individual variation in A3G/F-mediated CTL escape during HIV infection, to gain mechanistic insights into the molecular and cellular pathways through which A3G/F-mediated mutations drive CTL escape, and compare these to known pathways of immune evasion for HIV and other viruses.

In the future, it will also be important to fully define the rare set of CTL epitopes that we found are either not transformed into escape variants, or conversely, are made more immunogenic by A3G/F-mediated mutations. Our work suggests that any vaccination strategy, whether eliciting CTL or antibody responses, can be rendered more effective if these “A3G/F-resistant” or “A3G-enhanced” epitopes are taken into consideration.
